# Does road safety cointegrate with socio-economic conditions in rich developing countries?

**DOI:** 10.1371/journal.pone.0341441

**Published:** 2026-01-23

**Authors:** Ibrahim Abdalla Alfaki, Michal Grivna, Mohamed El Sadig

**Affiliations:** 1 College of Business and Economics, United Arab Emirates University, Al Ain, United Arab Emirates; 2 Institute of Public Health, United Arab Emirates University, Al Ain, United Arab Emirates; Shanghai Ocean University, JAPAN

## Abstract

Despite significant progress in road safety in developed countries, it remains a persistent and critical challenge in the developing world. This study investigates the long- and short-term relationships between socio-economic conditions and road safety performance in affluent developing countries, using the United Arab Emirates (UAE) as a case study. Employing an autoregressive distributed lag (ARDL) cointegration error-corrected model with data from 1980 to 2024 (sourced from the UAE Federal Government, the World Bank, and UN World Population Prospects), the analysis examines the link between the road crash severity index (fatalities to total injuries) and core socio-economic variables—GDP per capita, unemployment rate, and population density—while controlling for traffic law enforcement via fines. The findings confirm a long-term equilibrium, with an error correction term indicating road safety adjusts to socio-economic shocks at a rapid annual rate of 60%. Granger-causality tests further establish that these socio-economic factors significantly influence road safety outcomes, a concern underscored by an identified upward trend in crash severity. We conclude that socio-economic conditions are a fundamental determinant of road safety, highlighting the necessity for policy interventions that move beyond traditional engineering solutions. Consequently, road safety must be reframed not solely as a transportation concern but as an integral objective of public health and socioeconomic policy, which requires a collaborative, multi-sectoral approach to forge a resilient, safe system.

## Introduction

Road crashes are a significant drain on resources and threaten public safety and economic sustainability. In many countries, road safety is a top priority and a significant concern. More countries are adopting the WHO’s Safe System Approach to create a road system that protects all users by focusing on safe roads, vehicles, speeds, and post-crash care while acknowledging human error [1]. In addition to traditional engineering and behavioural strategies, it’s crucial to address social and economic factors to enhance road safety. This approach acknowledges the complex relationship between road crashes and economic development [2]. Road crashes can impede economic development by removing productive workers from the workforce, disrupting supply chains, and imposing significant costs. Simultaneously, economic development can exacerbate road crashes by increasing motorization and altering transportation patterns if road safety measures fail to keep pace [3–5]. Addressing this complex relationship requires a comprehensive approach to road safety as an integral part of sustainable economic development.

Developing countries face higher rates of road crash-related deaths due to factors such as poor roads, old vehicles, inadequate legislation, and limited law enforcement. More than 90% of road traffic deaths occur in low- and middle-income countries [3]. Road traffic injuries are the leading cause of death among children and young people aged 5–29 years. These crashes result in long-term disability, diminished quality of life, and substantial social and economic costs, including medical expenses and lost productivity, which negatively impact population health and economic development (Centers for Disease Control and Prevention (CDC), [6]). For example, in Cameroon, the social costs of road traffic crashes impose a significant burden, amounting to USD 3.6 billion in 2018, nearly equivalent to 3.8% of the country’s GDP [7]. Without proper planning and implementation of road safety measures, economic growth and development in developing countries may lead to infrastructure changes, such as road network expansion and urbanization, posing new safety challenges [[2,3,8,9]].

Conversely, developed and industrialized countries have seen improvements in road safety owing to economic growth. Governments and private enterprises have invested in improving road infrastructure, vehicle safety technologies, and traffic management systems. These include wider highways, advanced driver assistance systems, and intelligent transportation networks. Studies show that countries with higher GDP per capita have lower traffic fatality rates [[2,9–14]].

The International Transport Forum (ITF) [15] examined the relationship between GDP and crash casualties in OECD countries, which are generally considered developed economies. Among the developed OECD members, out of eight studies, five (63%) found a positive correlation between GDP and casualties, while two (25%) reported a negative correlation. In developing countries, 11 of 14 (78%) studies reported a positive relationship, while three studies (21%) showed a negative relationship. The findings suggest that economic growth often leads to increased casualties owing to higher traffic volumes and riskier driving behaviours. However, the negative correlations, particularly in developed countries, may suggest that as economies mature, investments in road safety can lead to reduced casualty rates despite increased traffic. This demonstrates that implementing effective safety measures and public policies can prevent a higher GDP from causing more deaths. This highlights the need for smart investments in the safety infrastructure to mitigate the adverse effects of economic growth.

Employment levels influence traffic patterns through commuting behavior, while unemployment can affect vehicle maintenance owing to financial constraints and potentially lead to increased risky driving behaviours stemming from psychological stress. Numerous studies have found a correlation between higher unemployment rates and increased road crashes and fatalities [16–17]. Some studies did not find a significant link [18], whereas others [19–21] have suggested that unemployment may reduce road crash fatalities. Thus, the results for unemployment are mixed, and it is unclear which of these effects is dominant. This mixed situation is confirmed by the ITF OECD 2015 report, which analyzed 28 studies on the link between economic development, measured by unemployment rates, and road casualties. They found that 79% (22 studies) indicated a significant positive relationship, suggesting that higher unemployment correlates with increased road casualties. This may be due to a reduction in driving frequency, but also to riskier behaviour among drivers, or an increase in dangerous driving habits during economic downturns. Conversely, 14% of the studies showed a significantly negative relationship, indicating that higher unemployment could lead to decreased road traffic and fewer road crashes. Although economic downturns often decrease traffic volume, the psychological and behavioural effects of unemployment can lead to riskier driving behaviours. This complex interaction between economic conditions and road safety underscores the need for policies that consider both economic factors and behavioural shifts associated with changes in employment rates.

The interplay between socio-economic conditions and road safety reflects a complex web of relationships. A higher population density typically increases traffic congestion and interaction opportunities between road users, potentially increasing crash risks [22–23]. This may be partially offset by lower vehicle speeds in densely populated areas and by effective urban planning [3]. It is also important to note that higher densities may be associated with lower car ownership and increased use of public transportation, which can alleviate traffic congestion and improve road safety [24]. Education level influences risk perception, understanding of traffic rules, and decision-making capabilities while driving, with higher education generally associated with better compliance with safety regulations [25]. These factors interact dynamically; for instance, densely populated areas with high education and employment levels might experience heavy traffic but show better regulatory compliance and risk awareness, while economically disadvantaged areas might experience less traffic but potentially more severe road crashes owing to infrastructure limitations and reduced access to vehicle safety technologies.

It is essential to recognize that socio-economic factors influence road safety by interacting with various other factors, including traffic violations, infrastructure investments, and the behavior of individuals from different demographic groups. This can be explored using multiple types of statistics, including regression for cross-sectional data, time series analysis for trends such as autoregressive integrated moving average (ARIMA), auto-regressive distributed lag (ARDL), autoregressive and error correction models, and panel data analyses that combine data types. The ARDL and error correction models are different from traditional methods because they allow simultaneous examination of both short- and long-term relationships between variables. They accommodate varying integration orders of data, allowing researchers to explore the impact of socio-economic variables on road safety over time, providing a complete understanding of these linkages.

Although road safety has markedly improved in developed nations, it remains a critical and persistent challenge in the developing world, creating a pressing need to understand its underlying determinants beyond conventional engineering approaches. This study is motivated by the specific context of affluent developing countries, which experience a unique paradox of rapid economic growth coupled with deteriorating road safety. This dynamic is poorly captured by existing models, that focus on developed or less-developed countries. Its significance lies in reframing road safety not solely as a transportation issue but also as a core socio-economic and public health objective, thereby demanding a multi-sectoral policy response. The novelty of this work is its application of an ARDL cointegration model to the United Arab Emirates (UAE), a rich developing economy, to empirically establish, using a data set spanning the period from 1980 to 2024, a long-run equilibrium and rapid short-run adjustment between a novel crash severity index and key socio-economic variables—GDP per capita, unemployment rate, and population density—while controlling for enforcement, thus providing a new quantitative framework for policy intervention.

To this end, this study utilizes an autoregressive distributed lag (ARDL) cointegration and error-correction modelling approach to examine both the long-term equilibrium and short-term dynamic relationships, as well as the Granger-causal linkages, between socio-economic factors and road safety (proxied by a crash severity index). The remainder of this paper is organized as follows. The next section presents a literature review relevant to the objectives of the study. The subsequent methodology section describes the data, theoretical justification for the variables, and ARDL model specification. The results section outlines the analytical process, including a description of the variables involved, unit root testing, cointegration analysis, and causality tests, and presents the key findings. This paper concludes with a discussion and acknowledges the limitations of the study. Finally, concluding remarks that situate the results within a broader context are presented.

## Literature review

The relationship between socio-economic conditions and road safety, while well-established in developed countries, remains critically underexplored within the unique context of affluent, rapidly developing countries. This review synthesizes existing literature to delineate the specific gaps that our study addresses.

First, there is a consensus on the fundamental influence of socio-economic factors. For instance, Sohaee and Bohluli [26] and Bektas [23] confirmed, through advanced modelling techniques like polynomial regression and econometric models, that variables such as GDP, unemployment, and vehicle density correlate with accident rates. However, a significant limitation persists: these and similar studies often focus on short-term correlations or developed contexts, thereby overlooking the long-term dynamics and moderating role of policy enforcement, such as traffic fines.

Building on this foundation, several studies have employed the necessary longitudinal frameworks but revealed further methodological and contextual shortcomings. Nikolaeva [27] emphasizes the macroeconomic impact of safety investments but lacks a nuanced analysis of crash severity over time. Similarly, Li et al. [28] and Akinyemi [29] successfully utilized Autoregressive Distributed Lag (ARDL) models to demonstrate long-run socio-economic equilibria in China and Nigeria, respectively. Despite their contributions, their analyses remain limited. They primarily focus on accident frequency (e.g., total fatalities) rather than a normalized measure of crash severity and fail to incorporate enforcement controls, which are crucial for accurate policy inference.

This gap is particularly evident when examining research conducted in comparable economic contexts. Ali et al. [30] analyzed upper-middle-income countries. They found that rising GDP per capita was associated with increased road traffic fatalities, a relationship that was potentially mitigated by factors such as health expenditure and population density. Crucially, however, their study also omitted enforcement-specific variables, an omission they identified as a key limitation for transferability to countries like the UAE, where rapid growth may outpace institutional safeguards.

Furthermore, the focus on developed economies limits the applicability of the findings. Ahmed et al. [31] highlighted the role of proactive environmental strategies and socio-economic conditions in improving sustainability and road safety in developing countries through integrated policies and technology. Cespedes et al. [32] provided valuable insights into how demographic shifts and urbanization affect injury severity in Spain. Nevertheless, their descriptive, developed-world focus leaves a gap in understanding the causal, long-term socio-economic dynamics and role of policy interventions in a developing context.

Recent studies in the region underscore enforcement’s direct link to economic savings and crash outcomes. A 2025 study on Saudi Arabia, a neighboring Gulf Cooperation Council state with comparable development and road safety challenges, quantified that scaling up enforcement interventions like speed monitoring could prevent hundreds of fatalities annually, yielding savings equivalent to 0.35% of GDP [33]. Within the UAE, detailed analyses of accident data consistently identify specific traffic violations (e.g., distracted driving, tailgating) as the leading causes of severe crashes, directly linking enforcement of specific laws to critical safety outcomes [34,35].

In synthesis, the extant literature converges on the socio-economic road safety nexus but exhibits a coherent set of gaps: (1) a lack of long-term cointegration analysis in affluent developing nations; (2) an overwhelming focus on accident frequency instead of a standardized severity index; and (3) a critical omission of traffic law enforcement as a control variable.

Therefore, this study aims to bridge these gaps by investigating the long- and short-run relationships between core socio-economic variables (GDP per capita, unemployment rate, and population density) and road safety performance, measured by a severity index (fatalities to injuries), in the UAE from 1980 to 2024. Employing an ARDL cointegration framework, our analysis explicitly controls for traffic law enforcement via fines, thereby offering a clear, context-aware, and policy-relevant understanding of road safety dynamics in a high-income developing nation.

## Methodology

The study used a quantitative longitudinal design to analyze the UAE road safety time series from 1980 to 2024, employing a Severity Index and socio-economic variables (GDP per capita, unemployment, and population density) and controlling for enforcement via road traffic fines data. It involves log-transforming data, testing for stationarity using Augmented Dickey-Fuller tests, and applying an ARDL bounds testing framework for cointegration in the EViews 13 program. An error-correction model was estimated to assess short-term dynamics and long-term relationships, while Granger-causality tests explored the direction of influence between socio-economic factors and road safety outcomes.

### Measures and data sources

Departing from conventional metrics, we introduce a Road Crash Severity Index (SI) to address the significant gap in road safety research (Table 1). Whereas prior studies predominantly relied on crash frequency data (e.g., total fatalities), our SI variable—the ratio of deaths to total injuries— explicitly measured the severity of crash outcomes, indicating the relative risk and seriousness of road crashes in terms of human casualties. This measure is not only less susceptible to fluctuations in exposure data but also serves as a crucial indicator of the underlying risk of death in a crash. Consequently, the SI provides a powerful lens through which to evaluate the life-saving efficacy of a nation’s post-crash care and emergency response infrastructure, a dimension often overlooked in socioeconomic models of road safety. We extracted data for this index from the UAE Federal Government database (Bayanati [36], and Al Amir [37]). The study also included road safety indicators and socio-economic variables with high availability and acceptable quality from well-established secondary sources. Using the UAE as a case study, we obtained time-series data on the country’s GDP growth per capita, unemployment rate (the number of unemployed people as a percentage of the labour force), and population density (the number of people living in one square kilometre). These data were obtained from the World Bank Database (World Bank [38]) and the World Population Prospects 2024 database (UNDESA [39]). The variable “FINE” reflects the total fines paid by drivers for traffic violations in UAE dirhams (1 USD = 3.67 dirhams), serving to assess the influence of public behavior, attitudes, and law enforcement on road safety. Higher values indicate improved enforcement and enhanced road safety.

**Table 1:** The study road safety and socio-economic indicators, UAE data 1980–2024.

Indicator	Description	Data Source
Road safety	• Severity index (percentage of fatal to total crash injuries (SI))	Bayanati [36], and Al Amir [37]
Socio-economic	• Growth of domestic product (GDP) per capita (GDP)	World Bank [38]
	• Unemployment rate (UNEMP)	World Bank [38]
	• Population density, no. of people per square kilometer (DEN)	World Population Prospects 2024 database (UNDESA [39]
Enforcement of traffic laws	• Total annual fines paid for violations of traffic laws in UAE Dirham (FINE)	Bayanati [36]-Ministry of Interior, Police reports.

The introduction and literature review demonstrate a strong theoretical and empirical consensus on the interdependence between selected socioeconomic variables and road traffic crashes or casualties.

#### Analytical framework: The ARDL approach.

The Autoregressive Distributed Lag (ARDL) approach and error correction models (ECM) are effective cointegration methodologies that can facilitate the analysis of dynamic short- and long-term relationships between socio-economic variables and road safety indicators. The ARDL model is particularly useful because it allows the inclusion of variables of different orders of integration (I(0) or I(1)) without losing information and provides robust estimates, even in small samples [40]. The ARDL approach can effectively capture the instantaneous and delayed impacts of economic development on road safety outcomes by enabling simultaneous estimation of long-term equilibrium relationships and short-term dynamics. The error correction model complements this by indicating how quickly the variables return to equilibrium after a shock [41,42]. Compared to traditional time-series techniques, which often require the variables to be integrated in the same order and can suffer from issues of overparameterization, ARDL and ECM offer greater flexibility and efficiency, making them particularly advantageous for analyzing complex interactions in socio-economic studies.

Our current investigation tracked socio-economic and road safety indicators from 1980 to 2024. The key indicators used to monitor changes in socio-economic conditions in the UAE are annual GDP per capita (GDP), unemployment rate (UNEMP), and population density per square kilometer (DEN). We used the variable (FINE) to control for the impact of law enforcement on safety outcomes. Equation (1) illustrates the ARDL model that uses the road crash severity index (SI) as the dependent variable. We can formulate similar ARDL models using GDP, UNEMP, DEN, and FINE as dependent variables.


$$\begin{array}{cc} \Delta {lnSI}_{t}= & \beta_{\text{0}}+\sum_{i=\text{1}}^{p}\lambda_{i}\Delta {lnSI}_{t-i}+\sum_{i=\text{0}}^{q}\delta_{i}\Delta {lnGDP}_{t-i}+\sum_{i=\text{0}}^{r}\alpha_{i}\Delta {lnUNEMP}_{t-i}\\ & +\sum_{i=\text{0}}^{s}\nu_{i}\Delta {lnDEN}_{t-i}+\sum_{i=\text{0}}^{z}\zeta_{i}\Delta {lnFINE}_{t-i}+\varphi_{\text{1}}{lnSI}_{t-\text{1}}\\ & +\varphi_{\text{2}}{lnGDP}_{t-\text{1}}+\varphi_{\text{3}}{lnUNEMP}_{t-\text{1}}+\varphi_{\text{4}}{lnDEN}_{t-\text{1}}+\varphi_{\text{5}}{lnFINE}_{t-\text{1}}+\varepsilon_{t}, \end{array}$$ (1)

where lnSI, lnGDP, lnUNEMP, lnDEN, and lnFINE are the log-transformed road safety severity index, average GDP per capita, unemployment rate, population density, and total annual fines paid for traffic violations, respectively, with corresponding lag lengths of *p*, *q*, *r*, *s* and *z*. The difference operator is denoted as *Δ*. Here, *φ*_i_*s* represents the long-term coefficients of the model and $\lambda_{\text{i}}$, $\delta_{\text{i}}$, $\alpha_{\text{i},}$ $\nu_{i}$, and $\zeta_{i}$ represent the short-term dynamic coefficients. The white noise error term is denoted $\varepsilon_{t}$.

Equation (1) is estimated using the ordinary least squares (OLS) method to specify the joint significance of the coefficient of lagged variables ($\varphi_{i}s$) using the *F-test*, comparing the F-statistic to Pesaran et al.‘s critical values [40]. The optimal lag order is selected using the Schwarz information criterion (SC). The null hypothesis of no cointegration relationship between the variables is expressed as $H_{\text{0}}:\;\varphi_{\text{1}}=\;\varphi_{\text{2}}=\varphi_{\text{3}}=\phi_{\text{4}}=\varphi_{\text{5}}=\text{0}.$ Rejecting $H_{\text{0}}$ suggests the presence of a long-term relationship among variables.

If there is cointegration (i.e., a long-term relationship between socio-economic variables and road safety), the relationship between the variables is not a transient situation but rather a more stable one that can be recovered whenever there is a disturbance or shock. What is the speed of adjustment to long-term equilibrium after a deviation occurs in the short term? Consequently, we describe the Granger causality and error correction representation of the ARDL model as follows:


$$\begin{array}{cc} \Delta {lnSI}_{t}= & \beta_{\text{0}}+\sum_{i=\text{1}}^{p}\lambda_{i}\Delta {lnSI}_{t-i}+\sum_{i=\text{0}}^{q}\delta_{i}\Delta {lnGDP}_{t-i}+\sum_{i=\text{0}}^{r}\alpha_{i}\Delta {lnUNEMP}_{t-i}+\sum_{i=\text{0}}^{s}\nu_{i}\Delta {lnDEN}_{t-i}\\ & +\sum_{i=\text{0}}^{z}\zeta_{i}\Delta {lnFINE}_{t-i}+\;\varphi {ECT}_{t-\text{1}}+\varepsilon_{t} \end{array}$$ (2)

${ECT}_{t-\text{1}}$ is the lagged OLS residual is obtained from the long-term model (${lnSI}_{t}=\beta_{\text{0}}+\beta_{\text{1}}{lnGDP}_{t}+\beta_{\text{2}}{lnUNEMP}_{t}+\beta_{\text{3}}{lnDEN}_{t}+\beta_{\text{4}}{lnFINE}_{t}+\varepsilon_{t},\;{\;ECT}_{t-\text{1}}=\varepsilon_{t-\text{1}}={lnSI}_{t-\text{1}}-(\beta_{\text{0}}\mathrm{\backslash allowbreak}+\beta_{\text{1}}{lnGDP}_{t-\text{1}}$$+\beta_{\text{2}}{lnUNEMP}_{t-\text{1}}+\beta_{\text{3}}{lnDEN}_{t-\text{1}}+\beta_{\text{4}}{lnFINE}_{t-\text{1}})).$ The coefficient of ${ECT}_{t-\text{1}}$, φ, is the speed of adjustment to long-term equilibrium to ensure convergence towards long-term equilibrium, φ, should be negative and significant. The model was considered unstable and explosive if φ was positive. If φ is significant, this also means that the regressors Granger cause the dependent variable in the long term. The causal impact was measured by testing the (t-test) coefficients λ, δ, α, ν, and ζ. If there are several lags in the regressors, a joint F-test (Wald test) can be performed to infer the joint short-term causality.

## Results

### Assessing UAE road safety data

Table 2 summarizes the log-transformed variables used in the analysis (1980–2024), providing essential context for model interpretation. The statistics indicate that GDP per capita exists on a substantially higher logarithmic order of magnitude than the other indicators, while Population Density shows the greatest relative dispersion. The proximity of the mean and median for most variables suggests the log transformation has effectively normalized their distributions, meeting a key precondition for reliable regression analysis and meaningful comparison of variable effects.

**Table 2:** Summary statistics of log-transformed variables (1980–2024).

Variable	Mean	Median	Standard Devition	Range
Severity index (SI)	1.895	1.902	0.231	0.831
Growth Domestic Product (GDP) per Capita	10.933	10.929	0.275	1.062
Population Density (DEN)	3.991	3.935	0.778	2.385
Unemployment Rate (UNEMP)	0.510	0.457	0.251	1.152

**Fig 1:**
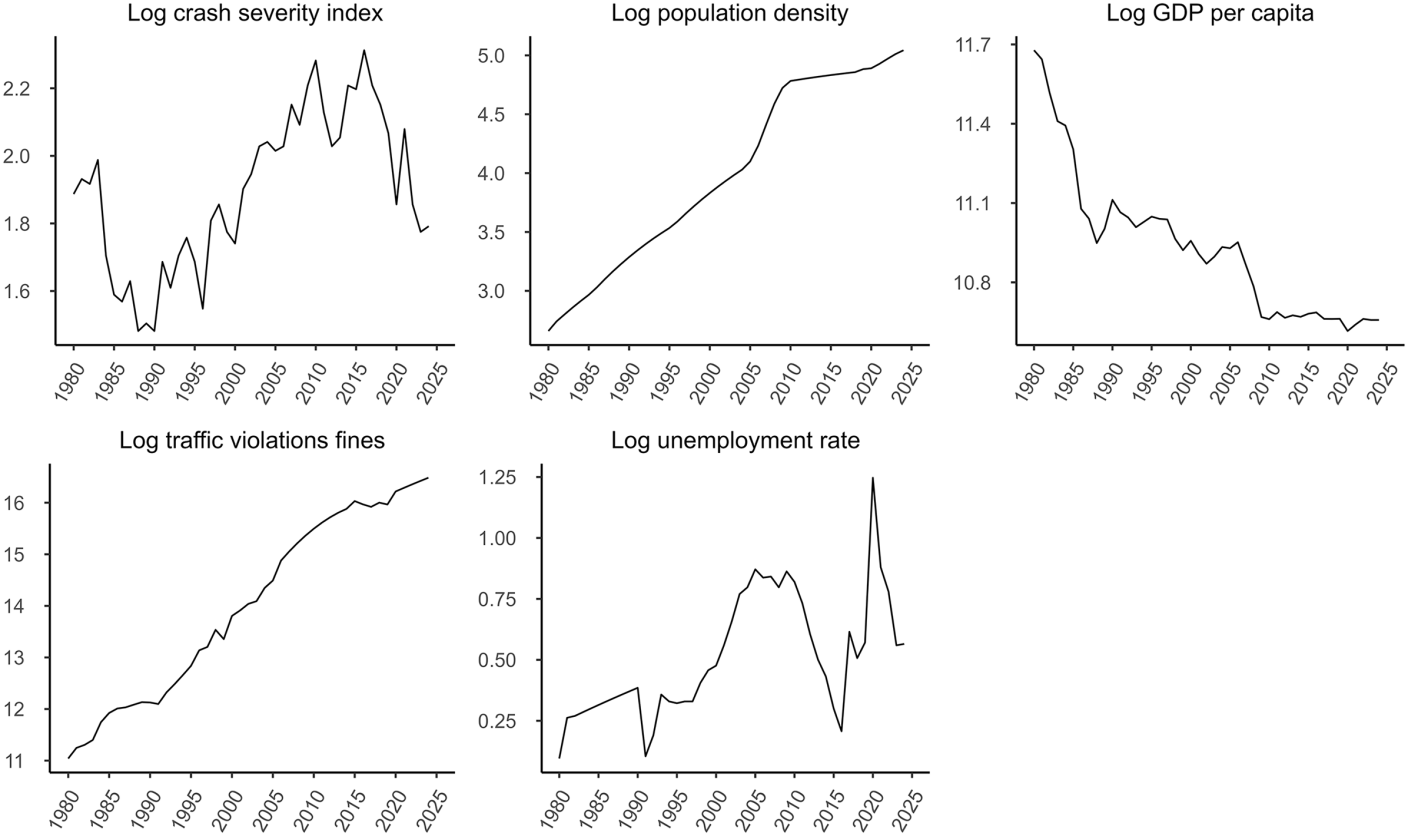
Log transformed road crash severity index, population density (people per square kilometers), and growth domestic product (GDP) per capita, total road traffic fines, and unemployment rate, UAE data 1980–2024.

As illustrated by the Road Crash Severity Index (Fig 1), the United Arab Emirates experienced a non-linear progression in road safety between 1980 and 2024. Historical data indicate an overarching fluctuating pattern, with significant temporal variations. Initial gains were evident prior to 1990; however, these were subsequently offset by a sustained period of deterioration lasting until approximately 2017. During this nearly three-decade span, the rising index values indicate an increasing severity of road traffic crashes, implying a growing proportion of crashes resulting in fatalities and an elevated relative risk to road users. We observed a notable reversal of this trend since 2017, suggesting a potential positive impact of recent policy interventions. As depicted in Fig 1, the population density of the UAE has increased over time. This can cause variations in road safety owing to factors such as increased pedestrian, cyclist, and motorcyclist congestion, and an increase in the volume of traffic. The UAE’s GDP per capita decreased from 1980 to 2024, reaching its lowest level in the aftermath of the 2008 global financial crisis, but has since begun a modest recovery, as shown in Fig 1. Increased GDP may have a positive impact on road safety because of investments in vehicle standards and road infrastructure. However, rapid economic growth and increased motorization can lead to challenges such as inadequate infrastructure, lack of road safety awareness, and a higher incidence of risky driving behaviours, all of which may have an impact on road safety outcomes. Since the mid-1990s, the UAE unemployment rate has increased slightly, dipping in 2016 before rising again through 2019 and dropping through 2024. Fig 1 also shows that the UAE has improved traffic law enforcement owing to tougher fines for traffic law violations.

### Assessing long- and short-term dynamic interaction among variables

The ARDL analysis and related tests were based on annual UAE time-series data from 1980 to 2024. lnSI, lnGDP, lnUNEMP, lnDEN, and lnFINE are the log-transformed road safety severity index for the UAE, average annual GDP per capita, unemployment rate, population density, and fines for traffic violations, respectively.

### Unit root test

Unit root analysis is the primary step in ARDL analysis, which verifies each series’ stationarity and order of integration to ensure that no variable is I(2) or greater. To satisfy the bounds test assumption for the SARDL models, each variable must be stationary at level I(0) or the first difference I(1). Table 3 shows that all series confirm I(1) using the augmented Dickey–Fuller (ADF) test.

**Table 3:** Results of the Augmented Dickey-Fuller (ADF) unit root tests.

Variable	Level			First Difference		
	t−value	p	*I*(0)	t−value	p	*I*(1)
lnSI	−1.6759	0.436	No	−7.5995	0.0000	*Yes*
lnGDP	−3.4045	0.016	yes	−4.7015	0.0004	*Yes*
*LUEMP*	−2.5259	0.116	No	−7.6395	0.0000	*Yes*
lnDEN	−1.6295	0.459	No	−3.7690	0.0063	*Yes*
*lnFINE*	−1.0873	0.712	No	−3.3776	0.0175	*Yes*

### ARDL bounds cointegration test

Based on the unit root test results (Table 3), which showed that no variable was I(2) or greater, the ARDL bound cointegration test was used to investigate the existence of a cointegration relationship between the UAE’s annual GDP per capita (lnGDP), unemployment rate (LUEMP), population density (lnDEN), traffic violations fines (lnFINE), and road safety as measured by the severity index (SI). The Schwarz information criterion (SC) recommends a maximum lag length of two, Equation (1). In contrast, the Hannan-Quinn (HQ) and Akaike Information criterion (AIC) suggest a maximum lag length of five. Based on these results, the ARDL analysis employed a maximum lag length of two suggested by the SC. The SC criterion is known as a consistent model selector, and tends to select the true model with the highest probability as the sample size increases (Table 4).

**Table 4:** ARDL bounds tests.

Dependent variable	Lag length	F-Statistic	Sig. level	Lower I(0) bound	Upper I(1) bound
${\text{F}}_{\text{SEV}}(\text{lnSEV}|\text{lnGDP},\text{lnUNEMP},\text{lnDEN},\text{lnFINE})$	2	5.93	5%	3.958	5.226
${\text{F}}_{\text{GDP}}(\text{lnGDP}|\text{lnSEV},\text{lnUNEMP},\text{lnDEN},\text{lnFINE})$	2	7.58	1%	5.376	7.092
${\text{F}}_{UNEMP}(\text{UNEMP}|\text{lnGDP},\text{lnSEV},\text{lnDEN},\text{lnFINE})$	2	8.60	1%	5.376	7.092
${\text{F}}_{\text{DEN}}(\text{lnDEN}|\text{lnSEV},\text{lnGDP},\text{lnUNEMP},\text{lnFINE})$	2	6.00	1%	5.376	7.092
${\text{F}}_{\text{FINE}}(\text{lnFINE}|\text{lnDEN},\text{lnSEV},\text{lnGDP},\text{lnUNEMP})$	2	3.59	10%	3.334	4.438

The ARDL bound test revealed a strong long-term link (cointegration) between socio-economic factors, traffic fines, and road safety when the log severity index (lnSI) was used as the dependent variable. The F-statistic = 5.93 exceeded the upper I(1)-bound critical value [40], as shown in Table 4 (p < 0.05). Similar significant long-term cointegration relationships (p < 0.01) were reported when GDP per capita, unemployment rate and population density were used as dependent variables. The bound test results are inconclusive (the test does not provide a clear answer) for the presence of a long-term relationship when traffic fines are used as a dependent variable, and the F-statistic lies between the I(0) and I(1) bounds (10%) (Table 4).

The results suggest a bidirectional but asymmetric relationship between crash severity and socioeconomic factors, in which crash severity responds more strongly to socioeconomic conditions (evidenced by the robust correlation when treated as the dependent variable, p < 0.01) than vice versa (weaker but still significant reverse correlation). This asymmetry aligns with empirical findings that lower GDP per capita and higher unemployment rates disproportionately increase crash severity owing to factors such as poor infrastructure, riskier driving behaviours, and limited access to safer vehicles [43,44]. Conversely, the feedback effect of crash severity on macroeconomic indicators (e.g., GDP, and unemployment) is subtler and often lagged, operating through indirect channels such as healthcare costs, lost productivity, and reduced economic activity in high-crash regions [44,45].

The persistence of significance in both directions nonetheless points to a systemic interdependence, consistent with theories of nonlinear socio-economic resilience [44]. For instance, studies show that while socioeconomic factors (e.g., education and income inequality) dominate short-term crash outcomes, severe crashes can exacerbate economic disparities over time, creating a reinforcing loop [43,45]. This aligns with Haddon’s framework, which emphasizes the interaction between humans, systems, and product performance in road safety [44].

### Granger causal impact of socio-economic conditions on road safety

Table 5 displays the estimated long-term coefficients of the ARDL model in terms of the significant cointegration between road safety (lnSI) and the UAE’s average annual GDP per capita. The estimated average GDP per capita (lnGDP) coefficient is statistically significant (p < 0.01) (Table 5). In the long-term, the average GDP per capita has a significantly positive impact on the severity of road crashes in the UAE. This implies that the increased level of economic growth in the country results in increased road crash fatalities and, thus, a deterioration in road safety, possibly due to increased population income and car ownership. A 1% increase in average GDP per capita results in an approximately 0.94% increase in road crash severity risk and a reduction in road safety in the long term. Ali et al. [30] also reported similar results. They found a positive correlation between GDP and road traffic fatalities in upper-middle-income countries, suggesting that economic growth leads to increased vehicle ownership and higher crash rates due to higher mobility and potential lapses in road safety. Table 5 also shows that a higher population density (lnDEN) is linked to a higher risk of fatal road crashes (p < 0.05), indicating less road safety. In the long term, a 1% increase in population density led to a 0.73% increase in the severity risk. Similar results have been reported by Ageli and Zaidan [46] and Li et al. [47].

**Table 5:** ARDL long and short-term estimated coefficients.

Dependent variable:lnSI_t_	Coefficient	Std error	t	P−value
***Long-term model coefficients***				
Constant	−9.7907^***^	2.6207	−3.7359	0.0006
${lnGDP}_{t}$	0.9363^***^	0.2237	4.1854	0.0002
${lnUNEMP}_{t}$	0.0798	0.1252	0.6375	0.5274
${lnDEN}_{t}$	0.7312^**^	0.3364	2.1735	0.0357
${lnFINE}_{t}$	−0.1078	0.1379	−0.7816	0.4391
***Error correction model coefficients***				
Constant	−2.4475^***^	0.4307	−5.6830	0.0000
*Trend*	−0.0539^***^	0.0089	−6.0547	0.0000
$\Delta {lnGDP}_{t}$	0.2451	0.2496	0.9821	0.3344
$\Delta {lnGDP}_{t-\text{1}}$	0.5740^**^	0.2465	2.3291	0.0273
$\Delta {lnGDP}_{t-\text{2}}$	0.7735^***^	0.2490	3.1067	0.0043
$\Delta {lnGDP}_{t-\text{3}}$	0.9387^***^	0.2433	3.8581	0.0006
ΔlnUNEMP	−0.1100	0.0798	−1.3784	0.1790
$\Delta {lnUNEMP}_{t-\text{1}}$	0.4178^***^	0.0850	4.9146	0.0000
$\Delta {lnUNEMP}_{t-\text{2}}$	0.1711^*^	0.0936	1.8283	0.0782
ΔLnDEN	−1.9298^**^	0.7666	−2.5174	0.0178
$\Delta {lnDEN}_{t-\text{1}}$	2.4704^***^	0.7362	3.3557	0.0023
ΔLnFINE	0.1124	0.1224	0.9124	0.3694
${ECT}_{t-\text{1}}$	−0.6028^***^	0.1025	−5.8831	0.0000
***Model Diagnostics:***	Test−statistics	p−value		
LM Serial correlation	2.3499	0.1189		
Heteroscedasticity (Breusch-Pagan-Godfirey)	0.7602	0.7110		
Normality (Jarque-Bera)	4.2120	0.1217		

Statistical significance: ^***^, ^**^, ^*^ corresponding to the 1%, 5%, and 10% significance levels.

The short-term dynamics of the economic and safety indicators were estimated using an error-correction model (Equation (2), set with automatic lag selection in EViews 13), which captures the adjustment process toward the long-run equilibrium relationship identified by the ARDL framework. The coefficient (−0.60) of the error correction term ${ECT}_{t-\text{1}}$ is negative and highly significant (p < 0.01) (Table 5). This determines the speed of adjustment and ensures long-term convergence to an equilibrium relationship. A negative and significant error correction term of −0.60 in the ARDL model indicates that road safety adjusts back to its long-term equilibrium at a relatively rapid rate after any disturbances driven by economic and behavioural factors. The analysis revealed Granger-causal relationships between socioeconomic factors and road safety outcomes, particularly crash severity, in the UAE. These findings highlight the importance of socioeconomic conditions in determining road safety performance and the need for targeted policy interventions to achieve sustainable improvements.

The lagged economic variables—GDP growth (∆lnGDP at lags 1, 2, and 3) and unemployment rate (∆lnUNEMP at lags 1 and 2)—exhibit statistically significant positive coefficients (p < 0.05 or 0.10, Table 5), indicating their delayed influence on crash severity in the UAE. This suggests that past economic fluctuations affect road safety as the system adjusts toward long-term equilibrium. When crash severity exceeds its equilibrium level, prior increases in GDP and unemployment further increase severity in subsequent periods. This delayed effect likely stems from gradual changes in infrastructure, travel demand, and driving behavior. The negative coefficient of −1.9298 for ${\Delta \text{lnDEN}}_{t}$ suggests that a higher population density in the current period reduces crash severity, likely due to factors such as slower traffic speeds, better emergency response proximity, or stricter urban traffic enforcement [48,49]. Conversely, the positive lagged effect (2.4704 for ${\Delta \text{lnDEN}}_{t-\text{1}}$ implies that higher density in the previous period increases severity, possibly reflecting delayed risks such as congestion-induced driver frustration, accumulated infrastructure strain, or temporal spillovers from increased exposure [50,51]. This dynamic aligns with studies showing that while dense areas benefit from immediate safety infrastructure, lagged effects may emerge from prolonged stressors, such as traffic volume or aging road networks [48,50]. Traffic fines, while theoretically exerting an immediate deterrent effect (as reflected in their negative coefficient in the long term, see Table 5), are statistically insignificant, implying limited long and short-term efficacy in mitigating crash severity.

These findings underscore the interplay between macroeconomic conditions and regulatory measures in shaping road safety outcomes. Lagged socio-economic performance exerts a persistent influence, whereas traffic fines—despite their intended role—appear ineffective in the short term (see [52]).

Short-term implications: Increased GDP per capita and lower unemployment rates from previous periods may strain vehicle safety standards, road infrastructure, emergency response systems, and traffic law enforcement, potentially, exacerbating crash severity.

Long-term Implications: Sustained economic growth could amplify crash severity through higher vehicle ownership, increased travel frequency, adoption of high-power vehicles, and riskier driving behaviours spurred by greater economic affluence.

## Model diagnostics and robustness checks

### Residual diagnostics and model stability

**Fig 2:**
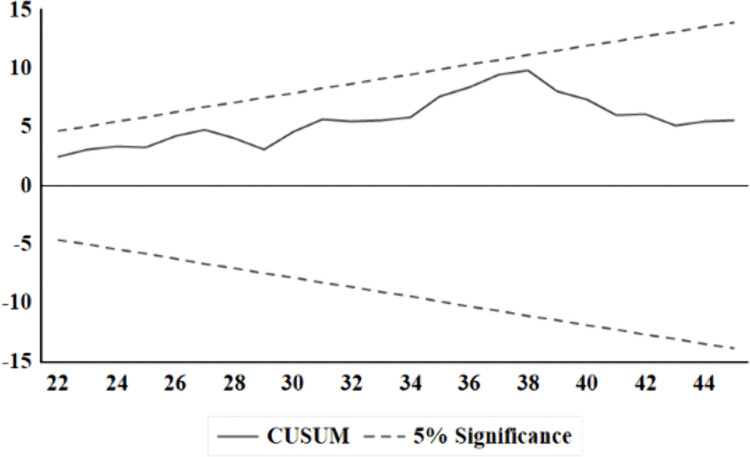
Cumulative total (cusum) test of recursive residuals. Dashed lines show critical bounds at the 5% significance level.

**Fig 3:**
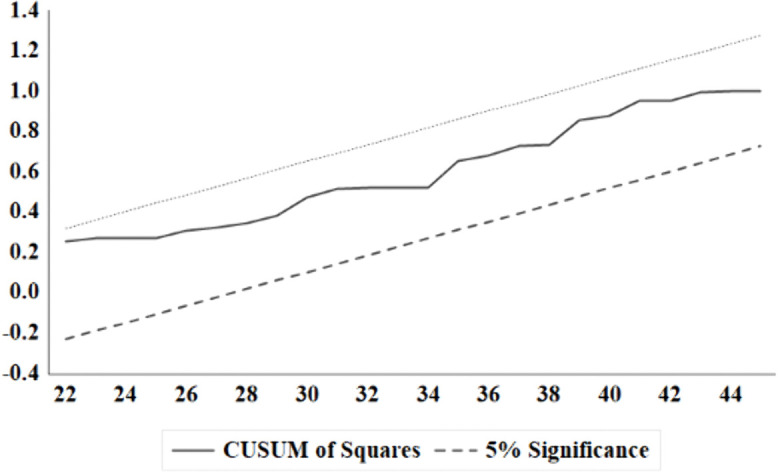
Cumulative total of squares (cusumsq) test of recursive residuals. Dashed lines show critical bounds at the 5% significance level.

Diagnostic tests on the residuals of the model confirmed its statistical adequacy. Standard tests for serial correlation (Breusch-Godfrey LM), heteroscedasticity (Breusch-Pagan-Godfrey), and non-normality (Jarque-Bera) fail to reject their respective null hypotheses at conventional significance levels, indicating that the residuals are well-behaved (Table 5). This result validates the model’s specification and the reliability of the inferred statistical significance. Complementing these findings, CUSUM and CUSUM-of-squares tests for parameter stability were conducted (Figs 2 and 3). Both statistics remain entirely within their 5% critical bounds, providing formal evidence of the model’s structural stability and the constancy of all estimated coefficients over the study period.

### Parameter stability and heterogeneity

**Fig 4:**
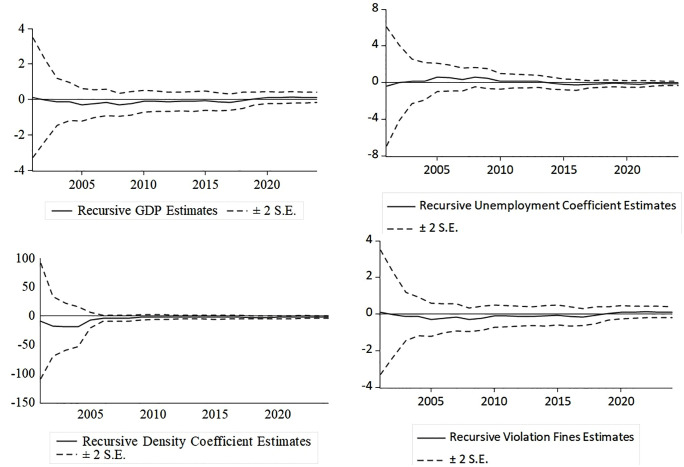
Recursive estimates of model coefficients.

Furthermore, the stability of the short-run parameters and error correction term was assessed through recursive estimation. The recursive coefficient plots for all explanatory variables—log GDP per capita, unemployment rate, population density, and traffic violation fines—revealed a pronounced pattern indicative of a robust parameter constancy (Fig 4). Throughout the sample period, the evolutionary path of each coefficient closely adhered to its full-sample estimate, with all values fluctuating narrowly within a band of ± two standard errors around zero. The absence of any discernible structural breaks or trending behavior demonstrates that the influence of these socio-economic factors on road safety outcomes remained stable and homogeneous over the analysis timeframe, thereby affirming the robustness of the estimated ARDL-ECM parameters against temporal heterogeneity.

## Discussion

### Interpretation of results

The empirical findings detail a complex, bidirectional interdependence between socioeconomic conditions and road crash severity in the UAE, characterized by a pronounced asymmetry in influence. The analysis robustly confirms that macroeconomic forces are the primary determinant of road safety outcomes. The significant long-term coefficient for GDP per capita (p < 0.01) suggests that economic growth paradoxically led to deterioration in road safety. This finding aligns directly with the work of Ali et al. [30] in upper-middle-income countries and reinforces the seminal hypothesis that rising income fuel increased vehicle ownership and mobility, potentially outpacing the development of safety regulations and infrastructure. This direct causal pathway is further reinforced by the Granger-causal impact of lagged GDP and unemployment, demonstrating that economic conditions from previous periods exert a persistent, delayed pressure on current crash severity, which is consistent with empirical observations that poorer infrastructure and riskier driving behaviours emerge from adverse socioeconomic conditions [43,44].

Conversely, the feedback effect of crash severity on the macroeconomic system, while statistically significant, operates through more indirect and lagged channels, a nuance that echoes the findings in extant literature [44,45]. The negative and highly significant error correction term (ECT = −0.60, p < 0.01) is pivotal here, indicating a rapid adjustment process that restores the system to its long-term equilibrium after a short-term shock. This dynamic suggests that the socioeconomic environment acts as the dominant attractor state, with crash severity behaving as the responsive variable. This supports the theoretical perspective that the economic impact of crashes is subtler, manifesting through accumulated healthcare costs and lost productivity rather than immediate macroeconomic shocks [43,45], a contrast to the immediate influence of the economy on crash risk.

The results regarding population density reveal a particularly intriguing temporal duality that both confirms and complicates prior research. The immediate, negative impact of current density (${\Delta lnDEN}_{t}$) on severity aligns with studies by Ageli and Zaidan [46] and others [48,49], which attribute this to slower traffic speeds and better emergency response proximity in dense urban areas. However, the subsequent positive effect of lagged density (${\Delta lnDEN}_{t-\text{1}}$) introduces a critical temporal dimension, suggesting that the long-term strains of congestion, infrastructure wear, and cumulative exposure eventually outweigh initial safety benefits. This delayed risk effect offers a nuanced explanation for the positive long-term coefficient observed in our model and that of Li et al. [28], suggesting that high density ultimately predicts higher severity, although through a delayed mechanism often overlooked in simpler cross-sectional analyses.

Ultimately, the model’s confirmation of systemic interdependence, consistent with theories of nonlinear socio-economic resilience [44], provides a framework for understanding the limited efficacy of isolated policy measures. The statistical insignificance of traffic fines underscores a finding consistent with the literature [52]: Regulatory deterrents are insufficient to counteract powerful socioeconomic undercurrents. This evidence strongly advocates for a shift in policy strategy, moving beyond reactive measures and towards proactive, system-level interventions that directly address the socioeconomic determinants of risk, such as strategic infrastructure investment and mandatory vehicle safety standards, to achieve sustainable improvements in road safety and break the reinforcing loop between economic disparity and crash outcomes [45].

### Theoretical implications

The most striking finding of this study is the positive long-term relationship between GDP per capita and crash severity, contradicting the conventional assumption that economic development inherently improves road safety [21]. Instead, the results support the hypothesis that rapid economic growth, particularly in developing high-income economies like the UAE, can lead to increased vehicle ownership, higher traffic volumes, and riskier driving behaviours before regulatory and infrastructural adaptations catch up [8]. This aligns with the “Smeed’s Law” paradox, which posits that fatalities initially rise with motorization before declining as safety measures mature [50]. The UAE’s mirrors the patterns observed in other upper-middle-income countries, where economic prosperity outpaces the institutional capacity for road safety [51].

Furthermore, Granger-causal relationships highlight the delayed effects of economic variables on crash severity, reinforcing theories of behavioural adaptation [53]. For instance, past GDP growth and unemployment rates continue to influence crash risks in subsequent periods, suggesting that economic booms may encourage risk-taking (e.g., speeding and distracted driving), while economic downturns could strain maintenance budgets and enforcement capabilities.

Our results illuminate the theoretically complex, dualistic nature of the impact of population density on road safety, revealing both immediate benefits and delayed risks [41,44]. This is evidenced by a significant long-term relationship where a 1% increase in density raises crash severity risk by 0.73% (p < 0.05), which is consistent with the existing literature [22,27]. The short-term dynamics further capture this temporal complexity: a contemporary increase in density reduces severity (coefficient: −1.93), likely due to slower traffic speeds and enhanced enforcement proximity [41,43], whereas a positive lagged effect (coefficient: 2.47) suggests a subsequent rise in risk from congestion-induced frustration, accumulated infrastructure strain, or exposure spillover [44,45]. This suggests that the initial protective effects of dense urban design are offset by latent, long-term, and lagged effects, necessitating proactive policies to mitigate these risks.

Rapid economic development in the UAE, while generating wealth, appears to negatively influence road user behaviour, fostering riskier practices such as distracted driving and speeding [21,54]. This effect is compounded by the uniquely diverse demographic landscape. The influx of a multinational workforce introduces a confluence of disparate driving cultures, training standards, and attitudes toward traffic laws, creating a fragmented driving environment that challenges uniform road safety adherence and contributes to increased crash and fatality rates [51,54].

### Practical implications

The results of this study have significant implications for policymakers, particularly in rapidly developing countries, where economic growth and road safety goals may appear at odds. First, the finding that traffic fines lack statistical significance in mitigating crash severity suggests that punitive measures alone are insufficient without complementary strategies such as infrastructure upgrades, public awareness campaigns, and stricter vehicle safety standards [55]. Given the UAE’s high GDP per capita, targeted investments in intelligent transportation systems (e.g., speed cameras and automated enforcement) and safer road designs (e.g., pedestrian zones and median barriers) could help mitigate the risks associated with increased mobility [56]. Dong et al. [57] highlighted that smart transportation innovations enhance efficiency and affect inequality through regional impacts and indirect factors, such as energy consumption and emissions efficiency. This suggests a multidimensional approach to road safety research in affluent developing countries, where technology and infrastructure interact with socio-economic conditions to influence outcomes.

Second, the delayed impact of the economic variables implies that road safety policies must adopt a proactive, forward-looking approach. For example, economic stimulus packages could include earmarked funding for road maintenance, while labour market reforms might address driver training and occupational safety in high-risk industries. UAE’s Vision 2030, which emphasizes sustainable development, presents an opportunity to integrate road safety into broader economic planning, such as linking urban density policies with transit-oriented development to reduce reliance on private vehicles [58].

Ultimately, the bidirectional relationship between socioeconomic factors and crash severity necessitates a systemic, multi-sectoral approach. Public health agencies, transport ministries, and economic planners must collaborate to address the root causes of unsafe driving (e.g., income inequality and inadequate public transit) while mitigating the long-term financial costs of crashes. Initiatives like the “Safe System” approach, which prioritize forgiving infrastructure and equitable access to safer mobility, could help break the reinforcing loop between economic disparities and road trauma (OECD [59]).

### Limitations and future research directions

The availability and quality of data on road crash casualties may impact the reliability and generalizability of the study. One limitation of this study was the lack of complete data. Only three variables were used to describe the socio-economic situation: GDP per capita, unemployment, and population density. It is important to note that higher levels of education may result in a better understanding of and adherence to traffic laws and regulations, as well as increased economic prospects. Other potential factors and confounding variables that may impact road safety and socio-economic conditions include social and cultural norms, attitudes toward road safety, and cultural behaviours. The use of the relative severity index as a dependent variable in this study has some limitations. This index may not account for all aspects of road crash casualties, such as those that incur no injuries or psychological consequences.

This limitations of the study present clear directives for subsequent scholarly inquiry. To construct a more comprehensive explanatory model, future research must integrate a broader set of variables, encompassing educational attainment, public health indicators, fleet composition, and infrastructural capital. The analytical resolution could be significantly enhanced by employing higher-frequency data, which would strengthen the Granger causality inferences concerning short-term dynamics. We recommend conducting a comparative study across GCC member states to evaluate the external validity of the results. Moreover, a mixed-methods approach is vital; qualitative investigations, including behavioral surveys and policymaker engagement, are necessary to provide a critical context for quantitative relationships and to interpret the complex role of socio-cultural factors and driver psychology.

## Conclusions

In conclusion, this study employed the ARDL cointegration error correction model to examine the long- and short-term relationships between road safety and socio-economic characteristics in developing countries. This study focuses on the UAE, using data from 1980 to 2024, as a case study to examine the impact of socio-economic factors and traffic fines resulting from traffic law violations on road safety, as measured by the road crash severity index (SI).

The study underscores that road safety in affluent developing nations is not merely a transport issue but also a socio-economic one, deeply intertwined with macroeconomic trends, urban development, and regulatory efficacy. While economic growth can inadvertently heighten crash risks in the short-to-medium term, strategic policy interventions can decouple prosperity from fatalities. Future research should explore granular regional disparities and the role of technology (e.g., electric vehicles and AI-driven traffic management) in moderating these relationships. For the UAE and similar economies, the path to safer roads is to harmonize economic ambitions with resilient and adaptive safety governance.
